# Evaluation of Two High‐Power Ablation Approaches in the Management of Typical Atrial Flutter: A Retrospective Study

**DOI:** 10.1111/anec.70128

**Published:** 2025-11-29

**Authors:** Hina Pervaiz, Farooq Hyder, Aimen Binte Moazzam, Fnu Abdullah, Abida Perveen, Jahanzeb Malik

**Affiliations:** ^1^ Department of Medicine Ibn e Seena Hospital Kabul Afghanistan

**Keywords:** atrial flutter, catheter ablation, cavotricuspid isthmus, high‐power ablation, irrigated catheter

## Abstract

**Objective:**

To compare the acute and long‐term outcomes of high‐power ablation for typical atrial flutter using a 4‐mm irrigated catheter (4‐IC) versus an 8‐mm non‐irrigated catheter (8‐NIC).

**Methods:**

We conducted a retrospective cohort study of 215 patients who underwent cavotricuspid isthmus (CTI) ablation between January 2019 and December 2024. Patients were divided into two groups based on the catheter used: 4‐IC (*n* = 113) and 8‐NIC (*n* = 102). Baseline, procedural, and follow‐up data were analyzed.

**Results:**

Both groups achieved 100% acute procedural success with no significant difference in CTI block rates. The 8‐NIC group had significantly shorter procedure duration (68.4 ± 15.2 vs. 77.4 ± 18.5 min, *p* < 0.001), reduced fluoroscopy time, and fewer lesions with shorter total RF delivery time. Periprocedural complications were rare and similar between groups. Over a mean follow‐up of 15.7 ± 7.2 months, atrial flutter recurrence occurred in 12.6% of patients, with no significant difference between groups (14.2% vs. 10.8%, *p* = 0.442). Rates of atrial fibrillation, pacemaker implantation, and continued anticoagulation were also comparable.

**Conclusion:**

Both ablation strategies are safe and effective, with the 8‐mm catheter offering greater procedural efficiency without compromising long‐term outcomes.

## Graphical abstract

To compare the acute and long‐term outcomes of high‐power ablation for typical atrial flutter using a 4‐mm irrigated catheter (4‐IC) versus an 8‐mm non‐irrigated catheter (8‐NIC). We conducted a retrospective cohort study of 215 patients who underwent cavotricuspid isthmus (CTI) ablation between January 2019 and December 2024. Patients were divided into two groups based on the catheter used: 4‐IC (*n* = 113) and 8‐NIC (*n* = 102). Baseline, procedural, and follow‐up data were analyzed. Both groups achieved 100% acute procedural success with no significant difference in CTI block rates. The 8‐NIC group had significantly shorter procedure duration (68.4 ± 15.2 vs. 77.4 ± 18.5 min, *p* < 0.001), reduced fluoroscopy time, and fewer lesions with shorter total RF delivery time. Periprocedural complications were rare and similar between groups. Over a mean follow‐up of 15.7 ± 7.2 months, atrial flutter recurrence occurred in 12.6% of patients, with no significant difference between groups (14.2% vs. 10.8%, *p* = 0.442). Rates of atrial fibrillation, pacemaker implantation, and continued anticoagulation were also comparable. Both ablation strategies are safe and effective, with the 8‐mm catheter offering greater procedural efficiency without compromising long‐term outcomes.

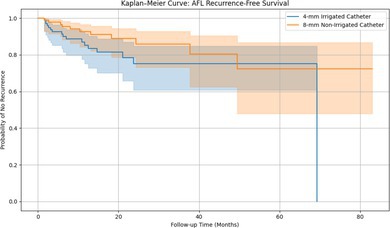

## 1 Introduction

Typical atrial flutter (AFL) is a common macro‐reentrant arrhythmia characterized by a counterclockwise circuit within the right atrium, dependent on the cavotricuspid isthmus (CTI) (Chun 2011; Tsai et al. 1999). This arrhythmia can lead to symptoms such as palpitations, fatigue, heart failure exacerbation, and increased thromboembolic risk. Radiofrequency (RF) catheter ablation targeting the CTI is the gold‐standard treatment, offering high acute success rates and low recurrence compared to medical therapy (Jais et al. 2000; Melo et al. 2007).

Traditional CTI ablation strategies have used standard 4 mm tip catheters with low‐to‐moderate power settings or large‐tip (8 mm) catheters, both to achieve bidirectional CTI block (Melo et al. 2007; Kasai et al. 2000; Cuesta et al. 2009; Da Costa et al. 2005; Scavée et al. 2004; Rodriguez et al. 2000). However, procedural efficiency and lesion durability may vary depending on catheter type, power delivery, and irrigation (De Ruvo et al. 2018). Recently, interest has grown in high‐power ablation strategies that reduce procedure time while maintaining safety and efficacy.

Two commonly used approaches in high‐power CTI ablation are the non‐irrigated 8 mm large‐tip catheter, which delivers high‐power without saline irrigation, and the 4 mm open‐irrigated catheter, which allows cooling and deeper lesion formation at higher powers (Melo et al. 2007; Kasai et al. 2000; Cuesta et al. 2009; Da Costa et al. 2005; Scavée et al. 2004; Rodriguez et al. 2000). The 8 mm catheter offers a larger surface area contact, potentially resulting in broader, shallower lesions, while the 4 mm irrigated catheter enables more controlled lesion depth and temperature regulation (Feld 2004a; Hsieh et al. 2002; Golian et al. 2020). Despite their widespread use, comparative data on acute efficacy, procedural characteristics, and long‐term outcomes between these two strategies is limited.

The objective of this study is to retrospectively compare acute procedural success and long‐term outcomes of high‐power CTI ablation using 8 mm non‐irrigated versus 4 mm irrigated catheters in patients with typical atrial flutter.

## 2 Methods

### 2.1 Study Design

This was a single‐center, retrospective cohort study conducted at Smart City Hospital in Pakistan. The study was approved by the institutional ethics committee (Reference no. SCH/25/002), and all procedures were carried out following institutional and ethical guidelines.

### 2.2 Study Population

We retrospectively reviewed the medical records of all patients who underwent cavotricuspid isthmus (CTI) ablation for typical atrial flutter between January 2019 and December 2024. Only patients who were treated with a high‐power ablation strategy using either an 8‐mm gold‐tip non‐irrigated catheter or a 4‐mm irrigated‐tip catheter were included. The choice of catheter was determined by the operating electrophysiologist at the time of the procedure. Based on catheter type, patients were categorized into two groups: the 8‐mm non‐irrigated catheter group (8‐NIC) and the 4‐mm irrigated catheter group (4‐IC). Patients with catheter crossover or additional ablation procedures (e.g., pulmonary vein isolation) were excluded.

### 2.3 Ablation Procedure

All procedures were performed under general anesthesia with continuous surface ECG and intracardiac electrogram monitoring. When available, fluoroscopy or a 3D mapping system (Ensite Velocity NavX, Abbott) guided the procedure. Vascular access was obtained via three femoral venous punctures under ultrasound guidance. A decapolar catheter was positioned in the coronary sinus, and a duodecapolar catheter was placed in the right atrium. Ablation was performed using either a 4‐mm open‐irrigated catheter (Therapy Cool Flex, Abbott) or an 8‐mm gold‐tip non‐irrigated catheter (AlCath LT G FullCircle 8 mm, Biotronik).

Radiofrequency energy was delivered point by point: 45 W for at least 30 s per lesion with the irrigated catheter, and 60 W for at least 30 s with the non‐irrigated catheter. A target impedance drop of 10–15 Ω guided lesion delivery. Ablation began at the tricuspid valve annulus and progressed toward the inferior vena cava. In patients in sinus rhythm, atrial pacing at 600 ms from the proximal coronary sinus guided lesion application.

Bidirectional CTI block was the procedural endpoint. Clockwise block was confirmed by reversed activation on the duodecapolar catheter during coronary sinus pacing, and counterclockwise block was assessed via sequential pacing and conduction delay measurement.

### 2.4 Follow‐Up

All patients underwent transthoracic echocardiography on the day following the procedure. Follow‐up data were retrieved retrospectively from outpatient records and referring cardiologists. Scheduled follow‐ups occurred at approximately 1, 6, and 12 months post ablation, including a 24‐h Holter ECG at 1 month. Long‐term follow‐up beyond 12 months varied based on the referring physician's discretion and the availability of documentation.

### 2.5 Outcomes

The primary efficacy outcome was defined as the achievement of bidirectional CTI block during the procedure. The primary safety outcome was any acute complication within 24 h, including pericardial effusion, vascular complications, hemodynamic instability, or stroke. Long‐term efficacy was defined as freedom from documented atrial flutter recurrence during follow‐up.

### 2.6 Statistical Analysis

Continuous variables were summarized as mean ± standard deviation, and categorical variables as counts and percentages. Group comparisons in the unmatched cohort were performed using Student's *t*‐test or chi‐square tests.

To account for potential selection bias in catheter choice, propensity scores were calculated using logistic regression models incorporating baseline characteristics such as age, sex, comorbidities, left ventricular ejection fraction, and prior ablation. Patients were matched 1:1 using nearest‐neighbor matching without replacement and a caliper width of 0.2 standard deviations of the logit of the propensity score. Covariate balance was evaluated using standardized mean differences, with values < 0.10 indicating acceptable balance.

In the matched cohort, continuous outcomes were compared using paired *t*‐tests, and binary outcomes using McNemar's test. Time‐to‐recurrence and survival curves were generated using Kaplan–Meier analysis and compared using the log‐rank test. Statistical significance was defined as a two‐tailed *p*‐value < 0.05. All analyses were performed using R software (version 4.3.3).

## 3 Results

### 3.1 Baseline Characteristics

A total of 215 patients undergoing cavotricuspid isthmus (CTI) ablation for typical atrial flutter were included, with 113 treated using a 4‐mm irrigated catheter (4‐IC) and 102 using an 8‐mm non‐irrigated catheter (8‐NIC). The mean age was 68.8 ± 10.2 years, with no significant difference between the two groups (*p* = 0.072). A higher proportion of women were treated in the 8‐NIC group (35.3% vs. 19.5%, *p* = 0.015). Other cardiovascular risk factors such as hypertension, diabetes, hypercholesterolemia, and tobacco use were comparable between the two groups (Table 1). The prevalence of atrial fibrillation (AF) history was significantly higher in the 8‐NIC group (46.1% vs. 31.9%, *p* = 0.038), whereas other comorbidities were similarly distributed.

**TABLE 1:** Baseline characteristics.

Variable	All Patients (*N* = 215)	4‐IC (*n* = 113)	8‐NIC (*n* = 102)	*p*
Age, years—mean ± SD	68.8 ± 10.2	67.6 ± 10.8	70.1 ± 9.3	0.072
Female sex—*n* (%)	58 (27.0)	22 (19.5)	36 (35.3)	0.015
Hypertension—*n* (%)	150 (69.8)	76 (67.3)	74 (72.5)	0.418
Diabetes mellitus—*n* (%)	69 (32.1)	35 (31.0)	34 (33.3)	0.737
Hypercholesterolemia—*n* (%)	134 (62.3)	70 (61.9)	64 (62.7)	1
Tobacco use—*n* (%)	92 (42.8)	48 (42.5)	44 (43.1)	0.924
Coronary artery disease—*n* (%)	70 (32.6)	38 (33.6)	32 (31.4)	0.757
Heart failure with reduced/mid EF (%)	61 (28.4)	31 (27.4)	30 (29.4)	0.753
Preserved EF—*n* (%)	35 (16.3)	15 (13.3)	20 (19.6)	0.206
History of atrial fibrillation—*n* (%)	83 (38.6)	36 (31.9)	47 (46.1)	0.038
Redo CTI ablation—*n* (%)	14 (6.5)	8 (7.1)	6 (5.9)	0.791
Pacemaker—*n* (%)	31 (14.4)	13 (11.5)	18 (17.6)	0.212
Sleep apnea—*n* (%)	34 (15.8)	20 (17.7)	14 (13.7)	0.436
COPD—*n* (%)	49 (22.8)	26 (23.0)	23 (22.5)	0.93
Thyroid disease—*n* (%)	28 (13.0)	11 (9.7)	17 (16.7)	0.139
History of stroke—*n* (%)	20 (9.3)	7 (6.2)	13 (12.7)	0.102
Chronic kidney disease—*n* (%)	58 (27.0)	28 (24.8)	30 (29.4)	0.461
History of cancer—*n* (%)	29 (13.5)	16 (14.2)	13 (12.7)	0.734
LVEF (%)—mean ± SD	53.5 ± 11.2	52.8 ± 12.0	54.3 ± 10.3	0.312

	All Patients	4‐IC	8‐NIC	*p*
**Medical Therapy—*n* (%)**				
*Medication*				
Class I antiarrhythmic	26 (12.1)	12 (10.6)	14 (13.7)	0.512
Class II (beta‐blockers)	140 (65.1)	74 (65.5)	66 (64.7)	0.901
Class III—Sotalol	6 (2.8)	4 (3.5)	2 (2.0)	0.691
Class III—Amiodarone	52 (24.2)	26 (23.0)	26 (25.5)	0.689
Class IV (calcium channel blockers)	12 (5.6)	8 (7.1)	4 (3.9)	0.368
Antiplatelet	32 (14.9)	14 (12.4)	18 (17.6)	0.276
**Anticoagulation—*n* (%)**				
*Drug*				
Apixaban	34 (15.8)	18 (15.9)	16 (15.7)	0.974
Dabigatran	22 (10.2)	10 (8.8)	12 (11.8)	0.475
Edoxaban	70 (32.6)	43 (38.1)	27 (26.5)	0.064
Rivaroxaban	55 (25.6)	27 (23.9)	28 (27.5)	0.559
Vitamin K antagonist	10 (4.7)	6 (5.3)	4 (3.9)	0.638
Low molecular weight heparin	6 (2.8)	3 (2.7)	3 (2.9)	1

### 3.2 Clinical Characteristics After Matching

After propensity score matching, baseline variables were well balanced between the 4‐IC and 8‐NIC groups (Table 2). There were no significant differences in age, sex, or major cardiovascular risk factors. The prevalence of prior AF, structural heart disease, and use of antiarrhythmic and anticoagulant therapies remained similar, indicating appropriate covariate adjustment.

**TABLE 2:** Clinical characteristics of the propensity score‐matched population.

Characteristic	All Patients (*N* = 215)	4‐IC (*n* = 113)	8‐NIC (*n* = 102)	*p*
Age, years—mean ± SD	68.7 ± 10.0	67.9 ± 10.2	69.6 ± 9.7	0.128
Female sex—*n* (%)	58 (27.0)	25 (22.1)	33 (32.4)	0.086
Arterial hypertension—*n* (%)	151 (70.2)	78 (69.0)	73 (71.6)	0.678
Hypercholesterolemia—*n* (%)	133 (61.9)	69 (61.1)	64 (62.7)	0.812
Diabetes mellitus—*n* (%)	74 (34.4)	38 (33.6)	36 (35.3)	0.79
Tobacco use—*n* (%)	95 (44.2)	50 (44.2)	45 (44.1)	0.99
Heart failure with reduced/mid EF—*n* (%)	64 (29.8)	32 (28.3)	32 (31.4)	0.623
Heart failure with preserved EF—*n* (%)	35 (16.3)	15 (13.3)	20 (19.6)	0.217
Coronary artery disease—*n* (%)	68 (31.6)	35 (31.0)	33 (32.4)	0.825
Atrial fibrillation history—*n* (%)	86 (40.0)	39 (34.5)	47 (46.1)	0.084
Redo CTI ablation—*n* (%)	14 (6.5)	7 (6.2)	7 (6.9)	0.836
Pacemaker—*n* (%)	33 (15.3)	14 (12.4)	19 (18.6)	0.214
Sleep apnea—*n* (%)	32 (14.9)	18 (15.9)	14 (13.7)	0.676
COPD—*n* (%)	52 (24.2)	26 (23.0)	26 (25.5)	0.684
Thyroid disorder—*n* (%)	27 (12.6)	12 (10.6)	15 (14.7)	0.354
Prior stroke—*n* (%)	20 (9.3)	7 (6.2)	13 (12.7)	0.095
Chronic kidney disease—*n* (%)	60 (27.9)	28 (24.8)	32 (31.4)	0.277
History of neoplasia—*n* (%)	29 (13.5)	15 (13.3)	14 (13.7)	0.928
LVEF (%)—mean ± SD	53.1 ± 11.0	52.7 ± 11.4	53.5 ± 10.6	0.583

	All Patients	4‐IC	8‐NIC	*p*
**Medical Therapy—*n* (%)**				
*Therapy*				
Class I antiarrhythmic	28 (13.0)	12 (10.6)	16 (15.7)	0.269
Class II (beta‐blocker)	141 (65.6)	74 (65.5)	67 (65.7)	0.98
Class III—Sotalol	6 (2.8)	3 (2.7)	3 (2.9)	0.943
Class III—Amiodarone	51 (23.7)	26 (23.0)	25 (24.5)	0.793
Class IV (CCB)	11 (5.1)	6 (5.3)	5 (4.9)	0.89
Antiplatelet	34 (15.8)	16 (14.2)	18 (17.6)	0.483
**Anticoagulation—*n* (%)**				
*Drug*				
Apixaban	35 (16.3)	18 (15.9)	17 (16.7)	0.874
Dabigatran	25 (11.6)	11 (9.7)	14 (13.7)	0.361
Edoxaban	72 (33.5)	45 (39.8)	27 (26.5)	0.037
Rivaroxaban	57 (26.5)	27 (23.9)	30 (29.4)	0.354
Vitamin K antagonist	10 (4.7)	6 (5.3)	4 (3.9)	0.61
Low molecular heparin	6 (2.8)	3 (2.7)	3 (2.9)	0.945

### 3.3 Procedural Outcomes

Procedural duration was significantly shorter in the 8‐NIC group (68.4 ± 15.2 min) compared to the 4‐IC group (77.4 ± 18.5 min, *p* < 0.001). Similarly, fluoroscopy time and total radiofrequency delivery time were significantly reduced in the 8‐NIC group (both *p* < 0.001). Despite fewer lesions and shorter RF duration, bidirectional CTI block and acute procedural success were achieved in nearly all patients (98.6% overall), with no difference between groups. The overall complication rate was low (1.9%), and there was no significant difference in periprocedural events between the two strategies (Table 3).

**TABLE 3:** Procedural characteristics.

Procedural parameter	All Patients (*N* = 215)	4‐IC (*n* = 113)	8‐NIC (*n* = 102)	*p*
Procedure duration, min—mean ± SD	73.2 ± 17.8	77.4 ± 18.5	68.4 ± 15.2	< 0.001
Fluoroscopy time, min—mean ± SD	11.7 ± 4.9	12.8 ± 5.2	10.5 ± 4.1	< 0.001
Use of 3D mapping—*n* (%)	106 (49.3)	59 (52.2)	47 (46.1)	0.377
Number of lesions applied—mean ± SD	21.1 ± 6.2	23.5 ± 5.7	18.4 ± 5.0	< 0.001
Total RF delivery time, sec—mean ± SD	742 ± 216	810 ± 205	663 ± 189	< 0.001
Impedance drop achieved (Ω)—mean ± SD	13.3 ± 2.5	13.5 ± 2.6	13.0 ± 2.4	0.128
Bidirectional CTI block achieved—*n* (%)	212 (98.6)	111 (98.2)	101 (99.0)	0.647
Acute procedural success—*n* (%)	215 (100.0)	113 (100.0)	102 (100.0)	—
Periprocedural complications—*n* (%)	4 (1.9)	2 (1.8)	2 (2.0)	1
Vascular access complication	2 (0.9)	1 (0.9)	1 (1.0)	1
Pericardial effusion	1 (0.5)	0 (0.0)	1 (1.0)	0.302
Transient AV block	1 (0.5)	1 (0.9)	0 (0.0)	0.328
Stroke	0 (0.0)	0 (0.0)	0 (0.0)	—

### 3.4 Long‐Term Outcomes

At a mean follow‐up of 15.7 ± 7.2 months, freedom from atrial flutter recurrence was slightly higher in the 8‐NIC group (89.2%) compared to the 4‐IC group (85.8%), though not statistically significant (*p* = 0.442). Rates of AF occurrence post‐ablation, redo ablations, and pacemaker implantation were also similar between groups. There was no significant difference in antiarrhythmic drug use, continued anticoagulation, or heart failure hospitalizations. All‐cause mortality was low (2.3%) and comparable (Table 4).

**TABLE 4:** Long‐term follow‐up of the propensity score‐matched population.

Follow‐up outcome	All patients (*N* = 215)	4‐IC (*n* = 113)	8‐NIC (*n* = 102)	*p*
Mean follow‐up duration, months ± SD	15.7 ± 7.2	15.3 ± 6.9	16.2 ± 7.5	0.31
AFL recurrence—*n* (%)	27 (12.6)	16 (14.2)	11 (10.8)	0.442
AF occurrence post‐ablation—*n* (%)	35 (16.3)	17 (15.0)	18 (17.6)	0.615
Redo CTI ablation—*n* (%)	11 (5.1)	6 (5.3)	5 (4.9)	0.893
Pacemaker implantation—*n* (%)	14 (6.5)	7 (6.2)	7 (6.9)	0.835
Antiarrhythmic drug use at last follow‐up—*n* (%)	47 (21.9)	26 (23.0)	21 (20.6)	0.681
Anticoagulation continued—*n* (%)	113 (52.6)	59 (52.2)	54 (52.9)	0.912
Hospitalization for heart failure—*n* (%)	12 (5.6)	6 (5.3)	6 (5.9)	0.85
All‐cause mortality—*n* (%)	5 (2.3)	2 (1.8)	3 (2.9)	0.652

### 3.5 Survival Analysis

**FIGURE 1:**
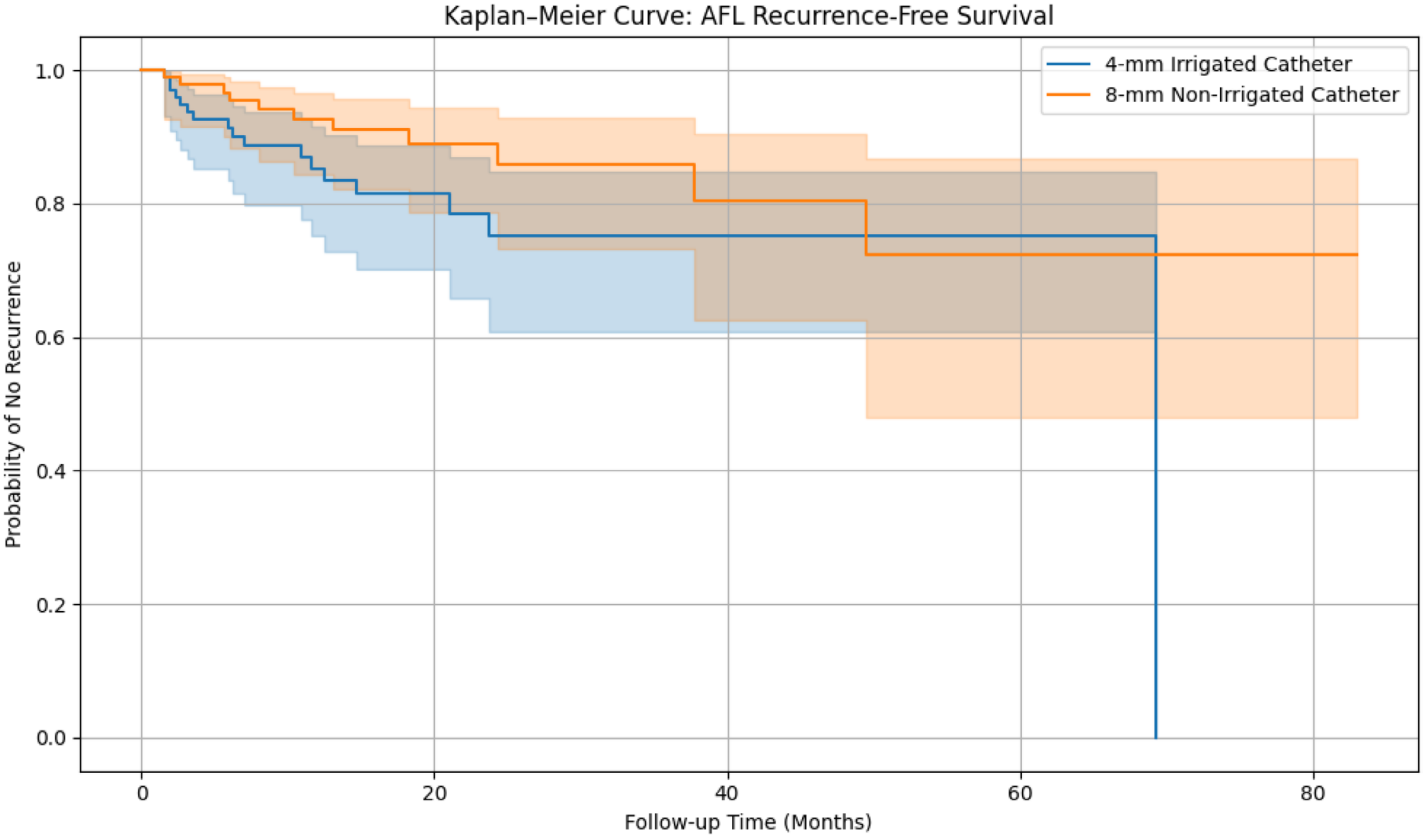
Kaplan–Meier analysis demonstrating long‐term freedom from typical atrial flutter. Log‐rank test *p*‐value: 0.1233.

Kaplan–Meier analysis revealed a non‐significant trend toward improved AFL recurrence‐free survival in the 8‐NIC group (Figure 1). Both groups maintained over 80% freedom from AFL at 24 months, with overlapping confidence intervals. The log‐rank test yielded a *p*‐value > 0.05, supporting the lack of statistically significant difference in long‐term efficacy.

## 4 Discussion

Our study demonstrates that high‐power radiofrequency ablation of the cavotricuspid isthmus (CTI) using both 8‐mm non‐irrigated and 4‐mm irrigated catheters achieves similarly high acute procedural success (~99%), procedural safety, and durable long‐term freedom from typical atrial flutter (AFL) (~85%–89%) over a mean follow‐up of approximately 16 months. These findings are in line with prior randomized and observational studies, which have shown comparable outcomes between catheter types in both efficacy and safety profiles (Tsai et al. 1999; Jais et al. 2000; Melo et al. 2007; Kasai et al. 2000; Cuesta et al. 2009).

Early randomized trials comparing irrigated‐tip catheters and 8‐mm tip catheters in typical AFL ablation reported similar success rates. Jais et al. noted no significant differences in acute CTI block success, procedure or fluoroscopy time between 4‐mm irrigated and conventional 4‐mm catheters, although irrigated tips demonstrated faster lesion delivery (Jais et al. 2000). More directly, Tsai et al. reported that 8‐mm tip catheters achieved higher rates of immediate bidirectional block (92% vs. 67%) with fewer applications and shorter procedural times compared to 4‐mm non‐irrigated tips (Melo et al. 2007). However, not all trials favored the large‐tip catheter; Kasai et al. and Cuesta et al. found no significant superiority in long‐term outcomes, reaffirming equivalent efficacy between the two designs (Kasai et al. 2000; Cuesta et al. 2009).

Meta‐analyses have generally concluded that both catheter platforms offer high efficacy and low complication rates for CTI‐dependent AFL ablation (Da Costa et al. 2005; Scavée et al. 2004). Our data echo this consensus. Procedural efficiency differed slightly: the 8‐mm catheter group had shorter mean procedure and fluoroscopy times, fewer lesions, and less total RF time, aligning with data from Tsai et al., Ventura et al. (2003) and Rodriguez et al. suggesting that larger tip catheters are more efficient in tissue targeting, particularly in patients with thicker isthmus anatomy (Melo et al. 2007; Rodriguez et al. 2000; Ventura et al. 2003).

A more recent multicenter registry comparing 8‐mm gold‐tip and irrigated‐tip catheters found no significant difference in cumulative RF time or procedural outcome at 6 months; however, fluoroscopy time was shorter with the 8‐mm catheter (De Ruvo et al. 2018). Our findings are consistent, although fluoroscopy time remained comparable—likely due to operator technique and mapping utilization. Overall AFL recurrence rates observed in our study fall within the 5%–12% range commonly reported in long‐term follow‐up analyses post‐CTI ablation (Feld 2004a; Hsieh et al. 2002).

Mechanistically, irrigated catheters enable deeper lesion formation with precise temperature control to reduce char formation and steam‐pop events (Tsai et al. 1999; Jais et al. 2000). Conversely, 8‐mm tips generate broader, shallower lesions effectively at high‐power, achieving similar lesion transmurality in the isthmus compared to irrigated tips (Melo et al. 2007; Da Costa et al. 2005). Given the variable anatomy of the CTI, with potential recesses and thicker muscular bridges, both catheter types appear effective across diverse tissue substrates, supporting operator preference based on procedural context (Da Costa et al. 2005; Scavée et al. 2004).

Our findings hold important implications for practice. They support clinician discretion in catheter selection, emphasizing factors such as catheter availability, cost, and experience, rather than perceived performance differences. The comparable long‐term outcomes also suggest that device choice need not be constrained by efficacy concerns in typical AFL ablation among experienced operators.

## 5 Clinical Implications

The findings of our study have direct and meaningful implications for clinical practice in the management of typical atrial flutter (AFL). First, the demonstrated equivalence in long‐term efficacy, acute procedural success, and safety outcomes between the 4‐mm irrigated catheter (4‐IC) and the 8‐mm non‐irrigated catheter (8‐NIC) suggests that both can be considered appropriate first‐line tools for CTI ablation. This flexibility enables electrophysiologists to base catheter choice on operator experience, cost, availability, and center‐specific workflow preferences, without compromising patient outcomes (Okishige et al. 2005; Shah et al. 1997; Schreieck et al. 2002; Rubín et al. 2017; Iori et al. 2014).

Importantly, the procedural efficiency observed with the 8‐mm catheter—characterized by shorter procedure and fluoroscopy times and fewer lesions—offers logistical advantages, particularly in high‐volume centers or in patients at increased risk from prolonged procedures, such as those with renal impairment or comorbid lung disease (Schreieck et al. 2002; Lewalter et al. 2017; Weiss et al. 2001; Hamaya et al. 2018). These advantages are well documented in previous randomized and real‐world studies (Schreieck et al. 2002; Lewalter et al. 2017; Weiss et al. 2001; Hamaya et al. 2018), and our study reinforces this efficiency benefit in contemporary practice.

Moreover, our comparable Kaplan–Meier survival curves highlight non‐inferiority in arrhythmia recurrence‐free survival over mid‐term follow‐up. This is particularly relevant for healthcare systems aiming to balance procedural efficiency, resource utilization, and patient outcomes. Clinical decision‐making regarding catheter selection can thus be individualized rather than protocol‐driven, potentially improving patient throughput and cost‐effectiveness without sacrificing clinical outcomes (Rubín et al. 2017; Ventura et al. 2004; Romero et al. 2017).

From a cost‐effectiveness standpoint, 8‐mm non‐irrigated catheters, which are generally less expensive and do not require fluid irrigation systems, may be preferable in resource‐constrained environments or in healthcare systems seeking value‐based solutions. Previous analyses have shown that for anatomically simpler lesions like CTI, the incremental benefits of irrigated catheters may not justify additional cost (Romero et al. 2017; Knecht et al. 2018; Kwon et al. 2020).

Finally, as new ablation technologies like pulsed field ablation emerge, this study reinforces that conventional RF ablation techniques remain highly effective for CTI‐dependent flutter. Given their safety profile and widespread availability, both 4‐IC and 8‐NIC remain valuable, durable tools in electrophysiology practice (Tsai et al. 1999; Melo et al. 2007; Feld 2004b; Johner et al. 2025; Pappone et al. 2011).

## 6 Limitations

This study has several limitations inherent to its retrospective, non‐randomized design. First, catheter selection was based on operator preference, introducing potential selection bias despite propensity score matching to balance baseline characteristics. Second, although procedural and follow‐up data were meticulously collected, the reliance on electronic medical records may have led to underreporting of minor complications or arrhythmia recurrences diagnosed outside the index institution. Third, follow‐up duration varied between patients, which may have influenced the assessment of long‐term outcomes. Fourth, the study was conducted at a single‐center with procedures performed by experienced operators, potentially limiting the generalizability of the results to other settings or less experienced centers. Lastly, use of 3D mapping was not standardized and may have influenced procedural efficiency outcomes independently of catheter type. These limitations underscore the need for prospective randomized trials to validate these findings.

## 7 Conclusion

The comparison of 4‐mm irrigated and 8‐mm non‐irrigated catheter strategies for typical atrial flutter ablation revealed similarly favorable safety and efficacy profiles. The 8‐mm catheter was associated with shorter procedure and fluoroscopy times. Long‐term freedom from atrial flutter was comparable between groups. Clinical outcomes, including recurrence and pacemaker implantation, did not significantly differ. These findings support the use of both catheter strategies, with the 8‐mm catheter offering procedural efficiency advantages.

## Author Contributions

**Hina Pervaiz:** writing, supervision, conceptualization, and methodology. **Farooq Hyder:** writing, validation, software, investigation. **Aimen Binte Moazzam:** formal analysis, data correction, supervision, methodology, and writing. **Fnu Abdullah:** project administration, writing, revision, investigation, software, and resources. **Abida Perveen:** supervision, writing, revision, methodology, software. **Jahanzeb Malik:** software, supervision, writing, literature search, revision, resources, methodology.

## Conflicts of Interest

The authors declare no conflicts of interest.

## Data Availability

Data sharing is not applicable to this article as no new data were created or analyzed in this study.
